# Gelseminum

**Published:** 1874-03-15

**Authors:** C. D. Hodge

**Affiliations:** Arkansas


### GELSEMINUM.

BY C. D. HODGE, M.D., OF ARKANSAS.

*From Southern Medical Record for Feb.*, 1874.

ON perusing an article in the November number of the *Record*, by Prof. Murry, of Baltimore, in which the gelseminum is favorably spoken of as an antiperiodic, I feel prompted to give some of the results of an experience of fifteen years or more with this article as a therapeutical agent.

Shortly after the febrifuge virtues of gelseminum were first accidentally discovered by a Mississippi planter, it was put forth as a nostrum in form of a branded tincture, under the cognomen of "Speed's Tonic;" and seeing among some of my patrons who had purchased and were using the medicine, that it *did* possess some very remarkable properties in controlling fever, by a little exertion I was fortunate enough to learn from one of the agents the plant; and as the vine grew abundantly around me, I lost no time in preparing a tincture, and instituting a series of trials, to get at its proper medical properties. Since that day I do not know that my case has been without a vial of tincture of gelseminum. My experience fully warrants me in endorsing all that has been claimed by Dr. Anderson, of North Carolina, and Prof. Murray, for this agent, as an antiperiodic. I have used it for years as such, in hundreds of cases of intermittent and remittent fevers, with as much satisfaction as ever I did with quinine, when relying upon that article alone. I usually combine the tincture with small doses of quinine, especially in the management of remittent cases. And just here let me assure my brother practitioners residing in malarious districts, that they can promptly arrest an ordinary uncomplicated case of chills with six grains of quinine and thirty drops of tincture of gelseminum, divided into, say, six doses — a dose every hour, beginning six hours preceding the chill-time. Just here I will add that a little preliminary medication, such as clearing the bowels, and if need be a mild address to the liver, is, I have found, more necessary than when using quinine alone. The tincture goes well with Fowler's Solution and tincture of iron, making it an efficient remedy for chronic cases, and as a chill preventive. In remittent cases this article can be used throughout the hot stage, in combination with the usual saline mixture, or any suitable diaphoretic, with the happy effect of shortening the febrile condition, and greatly curtailing the necessity for much quinine in the subsequent management.

Again, the value of gelseminum is not fully appreciated in treatment of neuralgias, especially those of an intermittent character. In these cases, it may be given in appropriate quantities, in combination with quinine, brom. pot. and mur. ammonia. With the latter, we think highly of it in the treatment of either acute or chronic sciatica. In apoplexy, where there is arterial excitement, with congestion, but no *rupture* of the vessels, the gelseminum shows itself an agent of signal potency. In uterine affections our experience is limited, but sufficient to justify the belief that this article will eventually become a remedy of no slight importance in that direction. In a case of tedious labor, where a rigid os uteri, or an unyielding perineum, offers the obstacle, we have only to apply an exhausted glass tumbler to the sacral spinal region, wait fifteen or twenty minutes, and let the patient have a commanding dose of the tincture — say twenty or twenty-five drops—and complete relaxation is almost sure to ensue very soon.

In treatment of irritable bladder, very favorable mention has already been made of this agent in some of the back numbers of the *Record*. It is also claimed as one of the "*very best remedies*," combined with opium, in dysentery. We can urge it as second to but few, *if any*, remedy, when associated with proper auxiliaries, in controlling recent gonorrhœa and acute ophthalmic affections. We will here casually say, owing to its peculiar physiological tendency to the organ, we believe the gelseminum will at no distant day take a prominent stand as an eye remedy.

The dose, in various diseases, may range all the way from two or three to twenty-five drops, according to urgency and other pointing of indications. But we think it is the better plan, particularly when given at short intervals, to use small doses, say four or five drops, and when its characteristic effects—muscular heaviness of the lids, perverted or double vision, etc. — begin to be manifested, lessen the dose, or rather prolong the spaces, or *suspend*, if effects are very marked.

We might draw much more from our somewhat extended experience in behalf of the tincture of gelseminum; but we set out for brevity, and must conform. However, before releasing our pen, we would say to those of our brethren who are disposed to give this article a trial, not to rely upon the *fluid extracts*, or any other preparation, except the *freshly-tinctured green root*, and the inner bark of the root at that. We are satisfied that a non-observance of this particular has impaired the confidence of many. We think the root-bark should be consigned to the alcohol within six hours after being taken from the ground. It should by all means be prepared in the month of September, or thereabouts, for it is comparatively worthless when made in the summer. If the above precautions are not strictly observed, you may expect disappointment in your trials. Our usual formula is six ounces of the finely-bruised bark of the root to a pint of diluted alcohol; let stand the usual time, firmly express, and filter.

